# Second language learning induces grey matter volume increase in people with multiple sclerosis

**DOI:** 10.1371/journal.pone.0226525

**Published:** 2019-12-23

**Authors:** Rainer Ehling, Matthias Amprosi, Benjamin Kremmel, Gabriel Bsteh, Kathrin Eberharter, Matthias Zehentner, Ruth Steiger, Noora Tuovinen, Elke R. Gizewski, Thomas Benke, Thomas Berger, Carol Spöttl, Christian Brenneis, Christoph Scherfler

**Affiliations:** 1 Department of Neurology, Clinic for Rehabilitation Münster, Münster, Austria; 2 Karl Landsteiner Institut für Interdisziplinäre Forschung am Reha Zentrum Münster, Münster, Austria; 3 Department of Neurology, Medical University of Innsbruck, Innsbruck, Austria; 4 Language Testing Research Group Innsbruck, Department for Subject Specific Education, University of Innsbruck, Innsbruck, Austria; 5 Department of Neuroradiology, Medical University of Innsbruck, Innsbruck, Austria; 6 Neuroimaging Research Core Facility, Medical University Innsbruck, Innsbruck, Austria; Heinrich-Heine-Universitat Dusseldorf, GERMANY

## Abstract

**Background:**

Grey matter volume (GMV) decline is a frequent finding in multiple sclerosis (MS), the most common chronic neurological disease in young adults. Increases of GMV were detected in language related brain regions following second language (L2) learning in healthy adults. Effects of L2 learning in people with MS (pwMS) have not been investigated so far.

**Methods:**

This study prospectively evaluated the potential of an eight-week L2 training on grey matter plasticity measured by 3T-MRI, L2 proficiency and health-related quality of life (HRQoL) in people with relapsing-remitting MS (pwMS, n = 11) and healthy, sex- and age-matched controls (HCs; n = 12).

**Results:**

Categorical voxel-based analysis revealed significantly less GMV bilaterally of the insula extending to the temporal pole in pwMS at baseline. Following L2 training, significant increases of GMV were evident in the right hippocampus, parahippocampus and putamen of pwMS and in the left insula of HCs. L2 training resulted in significant improvements of listening comprehension, speaking fluency and vocabulary knowledge in both pwMS and HCs. GMV increases of right hippocampus and parahippocampus significantly correlated with vocabulary knowledge gain and L2 learning was associated with a significant increase of HRQoL in pwMS.

**Conclusion:**

Our findings demonstrate distinct patterns of GMV increases of language related brain regions in pwMS and HCs and indicate disease-related compensatory cortical and subcortical plasticity to acquire L2 proficiency in pwMS.

## Introduction

Multiple sclerosis (MS) is a chronic and disabling disease of the central nervous system that predominantly affects young adults [1]. In addition to focal demyelinating white matter lesions, MS is characterized by progressive grey matter volume (GMV) loss [2]. The latter occurs early in the disease [3,4] and is closely correlated to cognitive impairment, a prevalent symptom affecting 43–70% of people with MS (pwMS) [5–7], that has a fundamental impact on health-related quality of life (HRQoL) [8]. While global GMV loss is commonly associated with cognitive impairment in MS [9,10], more recently, regional GMV reduction of distinct cortical areas was linked to frequently affected cognitive domains. Poor performance in sustained attention and information processing speed was correlated to volume loss in the auditory and premotor cortices [11], in the right inferior longitudinal fasciculus, the right inferior frontal gyrus and the left thalamus [12], and in the posterior cingulate and precuneus, caudate, putamen, and cerebellum [13]. In addition, visual memory impairment was associated with GMV loss of the splenium of the corpus callosum and the left inferior longitudinal fasciculus, while verbal fluency impairment was correlated to volume loss in the right insula and the left postcentral gyrus [13].

In healthy adults, second language (L2) learning was demonstrated to induce neuronal plasticity in language-related brain regions. In experimental training studies, the effect of L2 training over a minimum period of three months was associated with GMV increase of the right hippocampus, the left inferior and middle frontal gyrus and the left anterior temporal pole [14–16]. Neuroplasticity following L2 experience in these regions was not solely restricted to better L2 knowledge, but also associated with improvements in basic cognitive processes [17,18]. Cross-sectional studies revealed superior performance in memory and executive tasks of early and experienced bilinguals when compared to monolinguals [19,20]. Further, bilinguals were shown to exhibit a delay in the onset of dementia symptoms by four years compared to monolinguals [21], an observation that supports L2 learning as a training strategy for healthy cognitive aging [17]. However, whether L2 training has also the capability to induce advantageous effects on brain plasticity in pwMS has not been investigated so far.

In this explorative, prospective longitudinal study we aimed at clarifying (1) whether short L2 training induces similar brain plasticity in language-specific areas in pwMS and HCs; (2) whether pwMS are able to acquire a L2 as efficiently as age- and sex-matched healthy controls; and, (3) whether a potentially motivating activity like L2 learning improves HRQoL in pwMS.

## Methods

### Ethical statement, recruitment, in- and exclusion criteria

The study was approved by the ethics committee of the Medical University of Innsbruck, Austria (AN2015-0283 356/4.6). All participants gave written informed consent.

Participants were recruited prospectively between March 2016 and September 2017. Inclusion criteria for both groups were an age between 18 and 50 years, MRI eligibility, being right-handed and native German speaker. To account for homogeneous pre-existing English proficiency study participation was restricted to the levels A2 and B1 according to the Common European Framework for Languages [22].

PwMS were recruited prospectively at the Department of Neurology, Clinic for Rehabilitation Münster, and at the Clinical Department of Neurology, Medical University of Innsbruck. To account for a homogeneous MS cohort and minimize potential confounders in this exploratory trial, pwMS were included with a diagnosis of relapsing-remitting MS [23], a maximum disease duration of 10 years and a maximum EDSS of 4.0 [24]. Exclusion criteria comprised a relapse or corticosteroid therapy within 30 days prior to inclusion or during the L2 training intervention and a history of any psychiatric or neurological disorder other than MS. Since cognitive impairments may have an influence on L2 learning efficiency, normal overall performance in the Brief Repeatable Battery of Neuropsychological tests (BRB-N) was required [25]. The BRB-N has recently been recommended as a validated screening tool for cognitive deficits in MS [26] and refers to the four cognitive domains of short- and long-term memory, attention and executive function. To generate an outcome variable for overall cognitive function results of each subtest can be converted to a composite Z-score with a cut-off for abnormal performance being >1 standard deviation (SD) below the mean (<16th percentile).

To further account for a potential impact of anxiety and depression and/or MS-related fatigue on L2 learning, values in the Hospital Anxiety and Depression scale (cut-off value used for abnormal testing >7) and in the Würzburger Fatigue Inventory for MS scale had to be within normal limits (cut-off values indicating physical fatigue or cognitive fatigue >16 or >17 retrospectively, indicating any fatigue >32) [27].

Of 16 recruited pwMS, a total of five participants had to be excluded from the final analysis due to motion artefacts of MRI acquisitions (n = 2) and premature termination of the L2 training (n = 3).

Healthy individuals matched for age, sex, years of education and level of English proficiency were recruited by asking relatives or friends of already included pwMS and additionally by advertisement at the billboards of the two recruiting centres. Seven out of 19 recruited HCs had to be excluded from the final analysis. Three of them terminated the English training prematurely, MRI motion artefacts occurred in three participants and one subject could not be completed due to a dental titanium implant made during the interventional period.

### English language testing

Listening comprehension was measured by means of a bespoke version of the multilevel British Council APTIS Listening test [28,29], measuring candidates’ language abilities from A1 to C1 on the Common European Framework through a progression of tasks of increasing difficulty. The original 25-item listening test was extended by eight additional items from other versions of the test to increase the sensitivity of the instrument at the A2 and B1 levels. The speaking measure was operationalized as fluency demonstrated in speaking samples elicited via the APTIS Speaking test [28]. The speaking test comprised prompts of increasing difficulty delivered on a computer and candidates’ responses are recorded by the test system as they are spoken into a microphone. The total maximum speaking time on the test is 12 minutes. For the purpose of this study, speech rate was selected as the indicator of fluency. Speech rate is defined as the ratio of syllables to total speaking time (i.e. not counting pauses) and has been shown to be a robust marker of fluency in spoken language [30,31]. Sound files of the three standardized test tasks were analyzed using the PRAAT script Syllable Nuclei [32] to calculate the speech rate of each candidate performance [33]. Automated analyses using a tool like PRAAT allow for reliable and replicable measurements as manual coding and counting of pause lengths and syllables is highly error-prone. Vocabulary knowledge was tested using an adapted version of the Vocabulary Knowledge Scale [34]. 170 vocabulary items were included in the scale based on a thematic selection. For each item, participants had to choose one of the following response options: (1) I don’t know this word, (2) I have seen this word but don’t know what it means, (3) I have seen this word and think it means ___, (4) I know this word and it means ____). Responses were scored by trained rater, awarding 1 point if option 1 had been ticked, 2 points if option 2 had been ticked or a wrong translation/definition was given at options 3 and 4, as this indicated familiarity with the word but no knowledge of its meaning. 3 points were awarded if option 3 had been ticked and the word had been translated correctly. 4 points were given if option 4 had been selected and a correct translation/definition had been provided by the candidate. All three tests were administered in the same order pre and post L2 training intervention.

### Health-related quality of life

To enable for a comparison between pwMS and HCs HRQoL was assessed by the generic 36-item short form health survey (SF-36), which allows the discrimination of physical and mental health [35].

### MRI acquisition and processing

All MRI measurements were performed on a 3.0 Tesla whole-body MR scanner (Magnetom Skyra, Siemens, Erlangen, Germany) equipped with a twenty-channel head coil. All MRI acquisitions of the study were examined by a radiologist (EG), blinded to the diagnosis, upon its validity to be included to the study. The MRI protocol included 3D-T1-weighted, fluid-attenuated inversion-recovery, and PD-T2 weighted sequences. The MRI parameters for the axial T1-weighted 3D magnetization prepared rapid gradient echo (3D-MPRAGE) were set to TR = 1800 ms; TE = 2.22 ms; inversion time = 900 ms; number of excitations = 1; flip angle = 9°; field-of-view = 256×240mm; slices per slab = 192. T2-Turbo inversion recovery magnitude (TIRM) sequence parameters were set to TR = 10000 ms; TE = 90 ms; inversion time = 2500ms; slice thickness = 3 mm; voxel size = 0.9×0.9×3mm^3^, flip angle = 160° and field-of-view 240×195mm. The processing of MRI acquisition was controlled for quality before being entered into the statistical analysis. Initial default parameter values were used as a starting point for brain extraction. To provide appropriate results for each brain extraction, adjustments on parameters (i.e. coordinates for centre for initial brain surface sphere, fractional intensity threshold, and threshold gradient) were applied individually. FMRIB Software Library (FSL) was used to carry out brain extractions [36].

Registration of T2-TIRM and T1-MPRAGE was conducted by the FMRIB’s linear image registration tool FLIRT implemented into FSL software. An automated lesion growth algorithm as implemented in the lesion segmentation toolbox [37] for statistical parametric mapping (SPM, Wellcome Department of Cognitive Neurology, London, UK) [38] was used to (1) segment T2 hyperintense lesions and (2) fill in grey matter hypointense lesions [37]. The segmented lesions were reviewed by an expert (CS) and segmented lesion maps normalized to SPM space to exclude it from analysis.

To avoid a priori assumptions through region of interest analysis on brain areas of potential interests, grey and white matter volume and density measures were subjected to statistical parametric mapping (SPM, Wellcome Department of Cognitive Neurology, London, UK), a technique that objectively localizes focal changes of voxel values throughout the entire brain volume [38]. The software package SPM12 implemented in Matlab 9.2 (Mathsworks Inc., Sherborn, MA, USA) was used to preprocess and analyze MRI data. Voxel based morphometry (VBM) of grey and white matter compartment was performed by applying the standard version of the diffeomorphic anatomical registration using exponentiated lie algebra toolbox implemented in SPM12 to have a high-dimensional normalization protocol [39].

Segmented and modulated images were transformed from the study-specific diffeomorphic anatomical registration space into Montreal Neurological Institute space and smoothed by a Gaussian kernel of 8×8×8 mm in order to accommodate inter-individual anatomic variability and to improve signal to noise ratios for the statistical analysis. A masking threshold of 10% of the lower image signal was applied to reduce signal noise. For VBM analysis, age and total intracranial volume were entered as covariates. MRI acquisitions were processed on a Dell Studio XPS 435 T workstation with 8 cores, each with a 2.93 GHz Intel 7 processor.

### English language training

The intervention consisted of an eight-week English language training of three hours per week in a typical classroom setting. The training was designed to meet the needs of adult learners of the A2 and B1 levels of the Common European Framework for Languages [22]. It was structured around six topics that were considered relevant for everyday life and travel (e.g., shopping, directions) and focused on speaking, listening and vocabulary. The course followed a communicative approach to language teaching, focusing on meaning and use of language in comprehension and production. Fluency and successful understanding and conveying of meaning was foregrounded over accuracy drills. Vocabulary items were introduced at the form-meaning link level word by word first (e.g. through bilingual flash-cards), but were always embedded in an interactive classroom activity so that the vocabulary training happened in sentence-level and discourse-level contexts, as well as authentic situational tasks.

Self-study outside the classroom sessions was encouraged and participants received vocabulary lists and extra listening and speaking activities every week. The intervention was done in four consecutive cohorts and a medium group size of eight allowed for focusing on individual needs of participants. All cohorts were mixed in terms of pwMS and healthy controls, with the course instructor unaware of participants’ group membership. Classroom materials and activities remained identical between each cohort and the two teachers leading the total intervention employed various strategies in order to foster maximum learning (i.e. pair students with similar/dissimilar skills; repeat activities more than once; provide various scaffolding strategies). Table 1 details study procedures.

**Table 1:** Study procedures.

Screening	*Pre*-intervention testing	English language training	*Post*-intervention testing
- Inclusion/Exclusion criteria - English proficiency A2/B1* - Neuropsychological assessment (BRB-N), HADS, Würzburger Fatigue Inventory Scale**	- English proficiency testing (listening, speaking, vocabulary) - cMRI - SF-36	8 sessions in total, one session á 180 minutes weekly; classroom-setting; additional self-study	- English proficiency testing (fluency, listening, vocabulary) - cMRI - SF-36

Abbreviations: BRB-N, Brief Repeatable Battery of Neuropsychological tests; cMRI, cerebral magnetic resonance imaging; SF-36, 36-item short form health survey; HADS, hospitality anxiety and depression scale.

*according to the Common European Framework for Languages.

**evaluated in people with multiple sclerosis only.

### Statistical analysis

Demographic patient data are presented as absolute numbers and percentages, mean, SD, minimum and maximum or median and inter-quartile range (IQR). Differences between groups in categorical variables were evaluated using Fisher’s exact test. Numeric variables were analyzed by independent t-test or Mann Whitney-U test. Significance was based on a p-value of <0.05 and data was analyzed using SPSS Statistics-Version 22.0 (SPSS Inc., Chicago, IL, USA).

The obtained MRI datasets allowed for categorical comparisons of volume changes within the grey matter compartment. The cross-sectional between-groups and longitudinal within-groups comparisons of GMV were performed by a two-group (pwMS and HC) and two conditions (pre and post intervention) design implemented in SPM. Voxel-wise correlation analysis was carried out using a general linear model. GMV and the language testing scores of listening, speaking and vocabulary were entered into a design matrix. The relationship between MRI voxel values and parameters of cognitive performance were examined with t-contrast. Results from SPM analyses were corrected for multiple comparisons and the height threshold was set to p < 0.001 for ANOVA as well as correlation analyses and to p<0.05 for longitudinal within group analysis.

## Results

### Sample characteristics

Table 2 details baseline characteristics of the whole cohort. There was a pronounced female predominance (64%) and the majority of pwMS (73%) was on a disease modifying treatment (DMT). A median EDSS of 1.5 (interquartile range 1.0–2.0) indicated mild disability in the final MS cohort (n = 11). HCs (n = 12) were matched for age, sex, educational status and years of English lessons at school. Anxiety and depression scores were below the cut-off value >7 for abnormal testing and comparable between the MS cohort and the group of HCs. Scores on self-reported questionnaires were not indicative for the presence of cognitive or physical disease-related fatigue in the MS cohort. There were no relapses or signs of clinical progression in the MS cohort during the intervention. Baseline characteristics on the individual level are detailed in S1 Table.

**Table 2:** Baseline characteristics of study population.

Subject characteristics	people with MS	healthy controls	*p*-value
n	11	12	
Gender (females; males)	7; 4	10; 2	0.371^a^
Age (years)*	37.1 (7.8; 25.3, 46.3)	39.9 (8.8; 26.5, 49.3)	0.431^b^
Education (years)*	10.8 (1.5; 9, 12)	12.0 (3.3; 9, 17)	0.279^b^
English at school (years)*	8.6 (1.7; 6, 11)	8.6 (2.4; 5, 12)	0.933^b^
BRB-N sum score*	0.449 (0.882)	0.487 (0.649)	0.907^b^
Short-term memory	0.420 (0.942)	0.480 (0.629)	
Long-term memory	0.395 (0.764)	0.696 (0.412)	
Attention	0.007 (0.717)	0.109 (0.963)	
Executive function	0.514 (1.275)	-0.073 (0.696)	
Anxiety*	5.2 (3.5)	3.7 (2.3)	0.248^b^
Depression*	3.7 (3.2)	2.3 (1.9)	0.192^b^
Disease duration (years)*	3.3 (2.0; 0.7, 5.7)		
Expanded disability status scale**	1.5 (1.0–2.0)		
Disease modifying treatment (yes; no)***	8; 3		
Fatigue****	19.8 (10.8)		
Physical*	10.0 (6.8)		
Cognitive*	11.1 (6.2)		

Abbreviations: BRB-N, Brief Repeatable Battery of Neuropsychological Tests; a sum score >-1.67 refers to intact cognition [25].

^a^ Fisher’s exact

^b^ independent t-test.

* mean (standard deviation; minimum, maximum)

** median (interquartile range).

*** including dimethylfumarat (n = 1), glatiramer acetate (n = 1), interferon ß-1a 44 μg (n = 2), interferon ß-1a 30 μg (n = 1), natalizumab (n = 2) and teriflunomid (n = 1).

**** MS-related fatigue was evaluated using the Würzburger Fatigue Inventory scale [27]

### Second language proficiency

Prior to intervention, no significant between-group differences were observed in listening comprehension, speaking fluency and vocabulary scores in the MS cohort and HCs. After the L2 training listening comprehension, speaking fluency and vocabulary knowledge had significantly improved in the MS cohort (p = 0.001; p<0.001 and p<0.001 respectively) as well as in the group of HCs (p = 0.004; p = 0.042 and p<0.001 respectively; Table 3).

**Table 3:** Assessments of second language learning proficiency and health related quality of life.

	pwMS				Healthy controls					
	T1	T2	ΔT2-T1	*p-value* ^a^	T1	T2	ΔT2-T1	*p-value*^a^	*p-value*^b^	*p-value*^c^
Listening*	22.6 (5.3)	25.4 (4.4)	2.8 (2.1)	0.001	24.8 (4.6)	27.8 (2.6)	3.1 (2.9)	0.004	0.299	0.133
Speaking*	2.2 (0.4)	2.7 (0.4)	0.6 (0.2)	<0.001	2.2 (0.7)	2.6 (0.4)	0.6 (0.5)	0.042	0.854	0.536
Vocabulary*	2.9 (0.5)	3.6 (0.4)	0.7 (0.4)	<0.001	3.2 (0.4)	3.8 (0.1)	0.6 (0.3)	<0.001	0.071	0.082
HRQoL										
Physical health*	45.2 (10.5)	48.1 (14.7)	2.9 (6.1)	0.146	52.2 (6.6)	54.2 (5.1)	0.9 (3.1)	0.399	0.080	0.234
Mental health*	46.6 (14.2)	51.7 (12.4)	5.1 (5.5)	0.011	55.0 (5.3)	55.8 (3.8)	-1.0 (2.5)	0.259	0.023	0.446

Abbreviations: HRQoL, health related quality of life evaluated using the 36-item short form health survey; pwMS, people with multiple sclerosis. T1, baseline; T2, end of study; ΔT2-T1 refers to differences between baseline and end of study investigations with positive values indicating improvement.

^a^ Mann Whitney–U test

^b^ independent t-test comparing pwMS and healthy controls at T1

^c^ independent t-test comparing pwMS and healthy controls at T2.

* mean (standard deviation).

### Health-related quality of life

At baseline, physical health evaluated using the SF-36 showed comparable values in the MS cohort and in the group of HCs. However, scores of mental health were significantly lower in the pwMS group when compared to the HC group (p = 0.023). While subjectively perceived physical health was not altered at the end of the L2 training intervention in both groups, scores of mental health had significantly increased in pwMS (p = 0.011; Table 3).

### Imaging data

**Fig 1:**
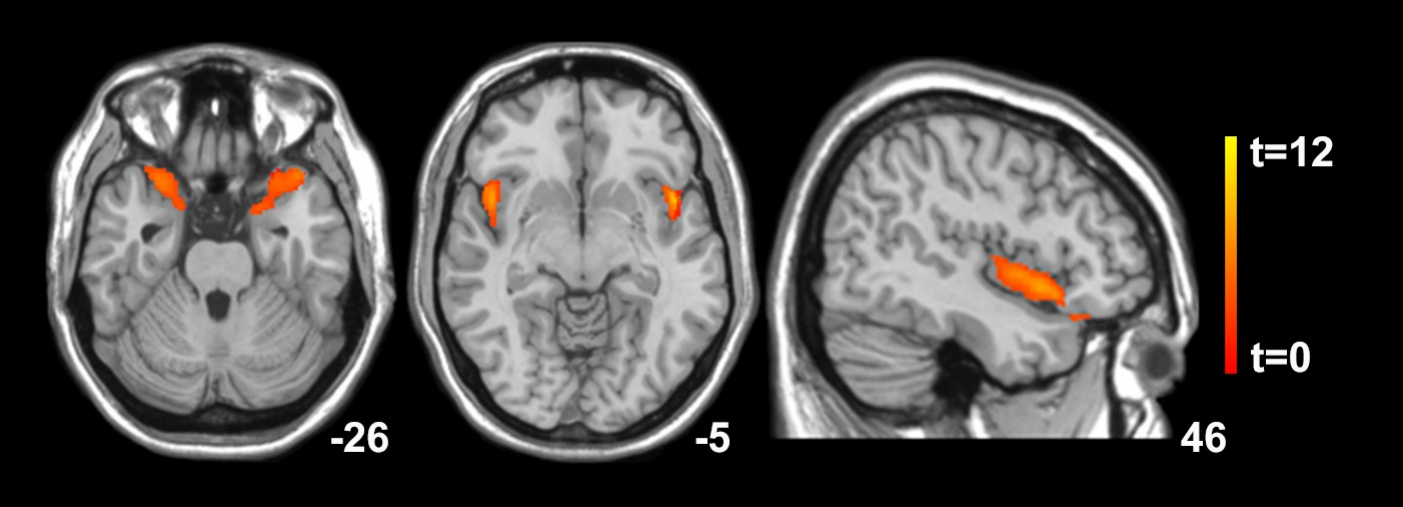
Statistical parametric mapping (*t*) intensity projection maps showing areas of significant decreases of grey matter volume (color code, yellow to orange) in a cohort of people with multiple sclerosis compared to healthy control subjects at baseline rendered on to a stereotactically normalized MRI scan in MNI space (z = -26 and -5; x = 46). The right side of the image corresponds to the right side of the brain.

**Fig 2:**
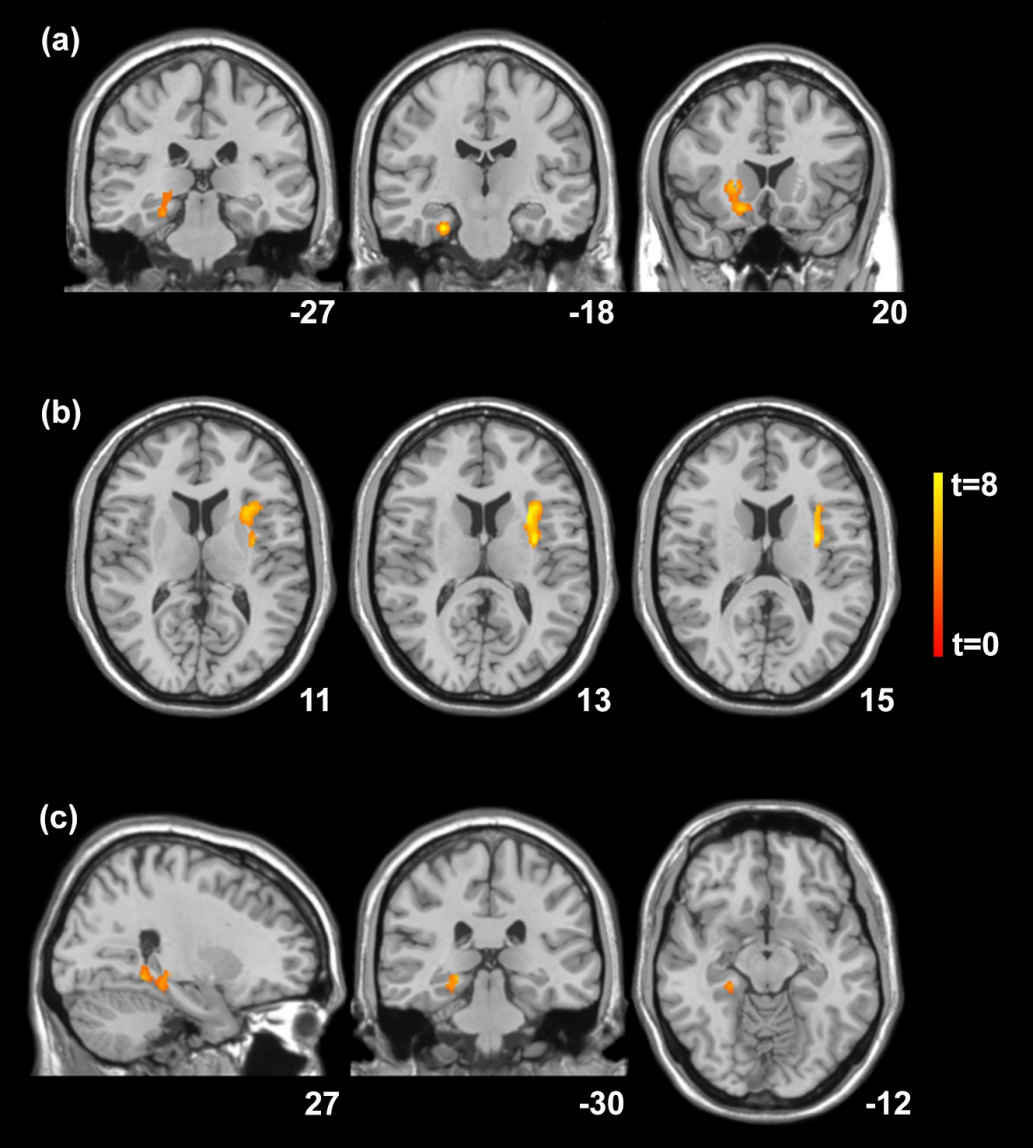
Statistical parametric mapping (*t*) intensity projection maps rendered onto a stereotactically normalized MRI scan, showing voxel cluster of increases of grey matter volume following second language training in (**a**) people with multiple sclerosis in MNI space (y = -27; -18 and 20; statistical significance thresholded at *p* < .001) and in **(b)** healthy control subjects in MNI space (z = 11; 13 and 15); **(c)** is showing areas of significant positive correlations of grey matter volume increase and vocabulary knowledge following second language training in a cohort of people with multiple sclerosis rendered on to a stereotactically normalized MRI scan in MNI space (x = 27; y = -30; z = -12).

Categorical voxel-based analysis revealed significantly less GMV bilaterally in the insula extending to the temporal pole of pwMS compared to the HC group at baseline (p<0.001; Table 3; Fig 1). The cumulative lesion volume of the entire MS cohort was 32.2ccm, with a total of 1.12ccm affecting the grey matter compartment. No grey matter lesions of the control group were identified visually and by the lesion segmentation toolbox. After the L2 training, significant GMV increases were localized to the right hippocampus and right parahippocampus (p = 0.015) and to the right anterior putamen of pwMS (p = 0.01; Table 3; Fig 2A). In the group of HCs, significant increases in GMV were found in the left insula following the English training intervention (p = 0.039; Table 4; Fig 2B). Spatial normalization-adjusted grey matter volume before and after the L2 training on the individual level are detailed in S2 Table and S1 Fig.

**Table 4:** Statistical parametric mapping findings showing the locations of significant changes of whole brain grey matter volume (VBM) following intervention; and correlation of increases in GMV with improvement in second language proficiency (vocabulary knowledge) in people with multiple sclerosis (pwMS) and healthy control subjects (HCs).

Cerebral regions	Cluster size (mm^3^)	X	Y	Z	*t*-value	*p*-value corrected at cluster level	Height threshold
Significant GMV decreases in pwMS at baseline compared to HCs							
Right Insula (BA 48)	3160	45	12	-5	12.4	0.001	0.001
Right temporal pole (BA 38)		26	21	-27	5.5		
Right parahippocampus (BA 28)		17	5	-24	4.0		
Left Insula (BA 48)	3542	-47	-11	-6	5.7	0.001	0.001
Left temporal pole (BA 38)		-30	20	-26	4.9		
Left parahippocampus (BA 28)		-21	9	-26	3.8		
Significant GMV increases in pwMS following intervention compared to baseline							
Right hippocampus	2008	24	-27	-5	4.9	0.015	0.001
Right parahippocampus (BA30)		27	-18	-26	9.3		
Right anterior putamen	2194	23	20	2	7.4	0.01	0.001
Significant GMV increases in HCs following intervention compared to baseline							
Left Insula (BA 48)	1542	-32	-2	15	7.8	0.039	0.001
Significant positive correlations of GMV increases and second language proficiency (vocabulary knowledge) in pwMS							
Right hippocampus	844	21	-29	-6	8.6	0.031	0.010
Right parahippocampus (BA 20, 37)		27	-30	-12	5.0		

XYZ coordinates mark the peak t-value within each cluster reported in Montreal Neurological Institute space coordinates. BA, Brodmann area.

GMV increase of the right hippocampus and parahippocampus was significantly correlated to the improvement in vocabulary knowledge in pwMS (p = 0.031; corrected at cluster level; Table 4; Fig 2C; S2 Fig); no correlations between listening comprehension as well as speaking fluency scores and MRI signal changes were evident. In HCs, no significant correlations were found between GMV changes and L2 proficiency scores.

## Discussion

The present study provides evidence for neuronal plasticity induced by L2 learning in a cohort of relapsing-remitting pwMS by demonstrating GMV increase of the right hippocampus, the right parahippocampus and the right anterior putamen.

Studies on L2 learning-induced GMV plasticity have so far been limited to studies in healthy adults [14–16,40–42], with only few of them following a prospective interventional design [14–16]. To the best of our knowledge, the present study is the first that has longitudinally investigated the effects of L2 learning on neuronal plasticity in people with a chronic neurological disease.

Although the investigated MS cohort was characterized by mild clinical disability and short disease duration, significant GMV reduction was already evident bilaterally of the insula extending to the temporal pole. This is in line with previous observations of progressive GMV decline in people with a clinically isolated syndrome or relapsing-remitting MS within three years after disease onset [4,43–45]. Despite significant GMV reduction at baseline, pwMS were as successful in learning an L2 in terms of listening comprehension, speaking fluency and vocabulary scores as their healthy counterparts. Moreover, L2 learning was accompanied by GMV increase in cortical (right hippocampal formation) and subcortical (right putamen) regions in pwMS. This finding is in accordance with previous studies of L2 learning in healthy adults, where MRI changes in the right hippocampus were reported after intense learning of vocabulary [46], a novel lexicon [47], or medical knowledge [48]. The right-hemispheric GMV increase in this context may represent a plasticity driven mirror-reverse of the traditional hippocampal domain-specificity, whereby language coded information is preferentially processed by the left, and visuo-spatial information by the right hippocampal formation [49]. The putamen [50], the caudate [51] or both [52] have been shown to be involved during language tasks, suggesting that basal ganglia structures may support L2 learning via visual imagery, verbal semantics and memory, language control and the management of simultaneity [53]. Similarly to the hippocampal formation, the finding of right putaminal GMV increase in our cohort of pwMS are hence suggestive of the involvement of the basal ganglia in plastic right hemisphere language-related networks. The activation of additional perilesional brain regions as well as cortical reorganization during task performance could have served as compensatory mechanism for pwMS in order to maintain cognitive functioning at an early stage of MS [54,55]. Since the present study focused on morphometric changes no conclusions can be made on the underlying functional mechanisms. However according to the significant correlation of the GMV increases of the hippocampus and the parahippocampus and the improvement in acquiring new vocabulary knowledge it is tempting to consider that L2 learning might serve as a trigger to enhance neuronal plasticity in pwMS at this early stage of the disease [56]. In this respect, further studies investigating whether L2 learning and especially the improvement of vocabulary knowledge could protect pwMS from the development of disease-related cognitive impairment, are needed [57,58].

Following L2 training, GMV increase was evident in the left insula in HCs, a brain region particularly involved in the motor control of speech production [59,60]. Although the functional implication of this finding is unclear, it might indicate cortical plasticity following the fostered use of active English conversation and articulatory processing during L2 training. PwMS in the present study did not exhibit a significant change in this particular brain region, which is most probably not only due to the pre-existent GMV decline in the insula bilaterally but also to the frequent functional disconnection between the basal ganglia and cortical areas in relapsing-remitting pwMS [61]. In contrast, intact cortical structures have likely allowed HCs to acquire higher L2 proficiency without activating additional brain regions as found in the cohort of pwMS. Although experience dependent effects of L2 learning on hippocampal cortical thickness and volume was reported in healthy adults, these changes were observed with a long and intense training protocol of three hours per day for three months or longer together with the acquisition of a new, previously unknown language [15]. The use of English as L2, a language that has already been familiar to the study participants to some extent and an L2 training frequency of three hours per week for two months used in the present study might have been of too low intensity for inducing neuroplasticity in intact brain regions relevant for acquiring L2 proficiency in HCs.

A significantly lower mental health status was reported by pwMS when compared to HCs at baseline, a frequent finding that has been observed in comparable MS cohorts previously [62]. After the L2 training, mental health subscores of HRQoL had significantly improved in pwMS suggesting that the positive experience of being capable to enhance L2 proficiency might have increased self-efficacy and participation possibilities in a chronic neurological disease like MS.

We have to acknowledge the limitations of this explorative study. First, the small sample size and the mild disability of the investigated MS cohort and the absence of a control group with MS do not allow for the generalisation of the findings at this stage and validation in a larger cohort is warranted. Second, although the correlation of GMV increase in the right hippocampus and parahippocampus with better vocabulary knowledge is suggestive for its functional relevance, its causal association still has to be established by functional neuroimaging protocols or paradigms. In this context it needs to be stated that L2 learning comprises not only episodic, semantic, short term and procedural memory functions, but many others including syntax and lexico-semantic abilities, comprehension, prosodic and phonetic learning and executive functions such as language switching, lexical selection, attention and inhibitory functions [63]. Thus only specific neuropsychological and linguistic testing would have allowed to precisely assign the observed improvements based on proficiency testing to specific cognitive domains, which has to be addressed in future studies.

We have not observed GMV increase in classic language- or memory-related brain regions such as the perisylvian cortex or the hippocampal formation of the left hemisphere in our study which is most likely due to both, the accomplishment of L2 learning predominantly through networks and the well known methodological difficulties of a direct functional-anatomical mirroring of structural changes in neuroplasticity studies [64].

In conclusion, the present study provides evidence for significant GMV increases in pwMS and age- and sex-matched HCs following a short L2 training in different language-related brain regions, is suggestive for disease specific compensatory mechanisms to acquire equal L2 proficiency in pwMS. At an early stage of the disease, L2 learning is feasible and accompanied by an improvement in the mental health status of pwMS. These encouraging results prompt further investigation of the effectiveness of L2 learning on the improvement of distinct cognitive impairments in pwMS.

## Supporting information

S1 Table Baseline characteristics of study participants on the individual level. Data is presented as *mean (standard deviation) or ** median (interquartile range). Female to male ratio in pwMS: 7/4, female to male ratio in healthy controls: 10/2. Mean age of pwMS: 37.1 (SD 7.8), mean age of healthy controls 39.9 (SD 8.8). For de-identification age and gender are not presented on the individual level.
Fatigue was evaluated using the Würzburger Fatigue Inventory scale [27].
Abbreviations: BRB-N, Brief Repeatable Battery of Neuropsychological Tests; a sum score >-1.67 refers to intact cognition [25]; EDSS, expanded disability status scale; DMT, disease modifying treatment including dimethylfumarat (n = 1), glatiramer acetate (n = 1), interferon ß-1a 44 μg (n = 2), interferon ß-1a 30 μg (n = 1), natalizumab (n = 2) and teriflunomid (n = 1); pwMS, person with Multiple Sclerosis; hc, healthy control.
(PDF)

S2 Table Spatial normalization-adjusted grey matter volume (GMV) in the voxel cluster of the right hippocampus/parahippocamus and the right putamen of people with Multiple Sclerosis (pwMS) and in the voxel cluster of the left insula of healthy controls (hc) at baseline (T1) and following the intervention (T2) on the individual level. Abbreviations: GMV, grey matter volume; T1, baseline; T2, end of study; ΔT2-T1 refers to differences between baseline and end of study investigations with positive values indicating GMV increase; pwMS, people with Multiple Sclerosis; hc, healthy control; SD, standard deviation.
(PDF)

S1 Fig **Spatial normalization-adjusted grey matter volume (y-axis) on the individual level at baseline and follow-up investigation in the area of the (a) right hippocampus/parahippocampus and the (b) right putamen in people with Multiple Sclerosis and in the area of the (c) left insula in healthy subjects.** Cyan lines indicate the mean of GMV evolution.
(TIF)

S2 Fig Pearson’s correlation of mean grey matter volume change (y-axis) in the area of the right hippocampus/parahippocampus and change of vocabulary knowledge following second language learning in people with Multiple Sclerosis (p = 0.031; ρ = 0.74). (TIF)
