# Supplementary material for: Second language learning induces grey matter volume increase in people with multiple sclerosis
Source: PLoS One. 2019 Dec 23;14(12):e0226525. doi: 10.1371/journal.pone.0226525 (PMC6927643; doi:10.1371/journal.pone.0226525)
Supplement: S1 Table — Data is presented as *mean (standard deviation) or ** median (interquartile range). Female to male ratio in pwMS: 7/4, female to male ratio in healthy controls: 10/2. Mean age of pwMS: 37.1 (SD 7.8), mean age of healthy controls 39.9 (SD 8.8). For de-identification age and gender are not presented on the individual level. Fatigue was evaluated using the Würzburger Fatigue Inventory scale [27]. Abbreviations: BRB-N, Brief Repeatable Battery of Neuropsychological Tests; a sum score >-1.67 refers to intact cognition [25]; EDSS, expanded disability status scale; DMT, disease modifying treatment including dimethylfumarat (n = 1), glatiramer acetate (n = 1), interferon ß-1a 44 μg (n = 2), interferon ß-1a 30 μg (n = 1), natalizumab (n = 2) and teriflunomid (n = 1); pwMS, person with Multiple Sclerosis; hc, healthy control. (PDF) [file pone.0226525.s001.pdf]

**S1 Table. Baseline characteristics of study participants on the individual level.**

| | education (years) | English at school (years) | BRB-N sum score | anxiety and depression | disease duration (years) | EDSS | DMT | Fatigue |
|---|---|---|---|---|---|---|---|---|
| pwMS_01 | 9 | 10 | -0.393 | 3 | 4.8 | 0 | yes | 10 |
| pwMS_02 | na | na | 0.499 | 6 | 1.2 | 4.0 | no | 18 |
| pwMS_03 | 9 | 7 | -0.022 | 12 | 5.6 | 1.5 | yes | 0 |
| pwMS_04 | 9 | 8 | -0.608 | 14 | 1.4 | 1.0 | yes | 32 |
| pwMS_05 | 12 | na | 0.710 | 11 | 3.1 | 1.0 | yes | 16 |
| pwMS_06 | 12 | 10 | -0.051 | 2 | 1.3 | 2.0 | yes | 10 |
| pwMS_07 | 12 | 11 | 1.239 | 5 | 0.7 | 2.0 | yes | 31 |
| pwMS_08 | 12 | 8 | 0.457 | 8 | 2.6 | 0 | no | 21 |
| pwMS_09 | 9 | na | -0.514 | 1 | 5.7 | 3.0 | yes | 17 |
| pwMS_10 | 12 | 6 | 1.556 | 14 | 5.5 | 2.0 | yes | 31 |
| pwMS_11 | 12 | 9 | 2.071 | 14 | 4.8 | 1.0 | no | 32 |
| | 10.8 (1.5)* | 8.6 (1.7)* | 0.449 (0.882)* | 8.9 (6.1)* | 3.3 (2.0)* | 1.5 (1.0-2.0)** | yes/no = 8/3 | 19.8 (10.8)* |
| hc_01 | 12 | 5 | 0.200 | 7 | | | | |
| hc_02 | 12 | 8 | 1.531 | 6 | | | | |
| hc_03 | 9 | 10 | -0.533 | 1 | | | | |
| hc_04 | 9 | 11 | 0.739 | 1 | | | | |
| hc_05 | 9 | na | 0.602 | 11 | | | | |
| hc_06 | 17 | 6 | -0.187 | 10 | | | | |

| | | | | |
|---|---|---|---|---|
| hc_07 | 17 | 10 | 0.223 | 3 |
| hc_08 | 9 | 5 | 1.330 | 4 |
| hc_09 | 12 | 10 | 0.034 | 9 |
| hc_10 | 12 | 9 | 0.157 | 11 |
| hc_11 | 9 | 8 | 0.381 | 1 |
| hc_12 | 17 | 12 | 1.370 | 7 |
| | 12.0 (3.3)* | 8.6 (2.4)* | 0.487 (0.649)* | 5.9 (3.9)* |
