# Supplementary material for: Second language learning induces grey matter volume increase in people with multiple sclerosis
Source: PLoS One. 2019 Dec 23;14(12):e0226525. doi: 10.1371/journal.pone.0226525 (PMC6927643; doi:10.1371/journal.pone.0226525)
Supplement: S2 Table — Abbreviations: GMV, grey matter volume; T1, baseline; T2, end of study; ΔT2-T1 refers to differences between baseline and end of study investigations with positive values indicating GMV increase; pwMS, people with Multiple Sclerosis; hc, healthy control; SD, standard deviation. (PDF) [file pone.0226525.s002.pdf]

S2 Table. Spatial normalization-adjusted grey matter volume (GMV) in the voxel cluster of the right hippocampus/ parahippocamus and the right putamen of people with Multiple Sclerosis (pwMS) and in the voxel cluster of the left insula of healthy controls (hc) at baseline (T1) and following the intervention (T2) on the individual level.

	**GMV right para-/hippocampus**			**GMV right putamen**				**GMV left insula**		
**pwMS_#**	**T1**	**T2**	**ΔT2-T1**	**T1**	**T2**	**ΔT2-T1**	**hc_#**	**T1**	**T2**	**ΔT2-T1**
pwMS_01	0,4854	0,5081	0,0455	0,3975	0,4145	0,0170	hc_01	0,3852	0,3985	0,0134
pwMS_02	0,4540	0,4463	-0,0197	0,3111	0,3148	0,0036	hc_02	0,3603	0,3673	0,0070
pwMS_03	0,4489	0,4737	0,0396	0,3545	0,3727	0,0182	hc_03	0,4053	0,4188	0,0135
pwMS_04	0,3963	0,3978	0,0191	0,4172	0,4212	0,0040	hc_04	0,3550	0,3789	0,0239
pwMS_05	0,3869	0,3967	0,0262	0,3113	0,3278	0,0165	hc_05	0,3485	0,3667	0,0182
pwMS_06	0,4894	0,4708	-0,0228	0,4035	0,4270	0,0235	hc_06	0,3282	0,3376	0,0093
pwMS_07	0,5041	0,5133	0,0263	0,4190	0,4324	0,0134	hc_07	0,3935	0,3999	0,0065
pwMS_08	0,4282	0,4487	0,0350	0,3759	0,3849	0,0090	hc_08	0,3204	0,3407	0,0202
pwMS_09	0,4496	0,4838	0,0487	0,3854	0,4035	0,0181	hc_09	0,3249	0,3576	0,0327
pwMS_10	0,4612	0,4743	0,0284	0,3669	0,3862	0,0193	hc_10	0,3108	0,3187	0,0079
pwMS_11	0,4875	0,5056	0,0349	0,4254	0,4445	0,0192	hc_11	0,3874	0,4042	0,0168
							hc_12	0,3221	0,3317	0,0095
mean	0,4538	0,4654	0,0237	0,3789	0,3936	0,0147	mean	0,3535	0,3684	0,0149
SD	0,0380	0,0402	0,0239	0,0402	0,0420	0,0065	SD	0,0328	0,0323	0,0079

Abbreviations: GMV, grey matter volume; T1, baseline; T2, end of study; ΔT2-T1 refers to differences between baseline and end of study investigations with positive values indicating GMV increase; pwMS, people with Multiple Sclerosis; hc, healthy control; SD, standard deviation.
