# Supplementary figures and images for: Second language learning induces grey matter volume increase in people with multiple sclerosis
Source: PLoS One. 2019 Dec 23;14(12):e0226525. doi: 10.1371/journal.pone.0226525 (PMC6927643; doi:10.1371/journal.pone.0226525)

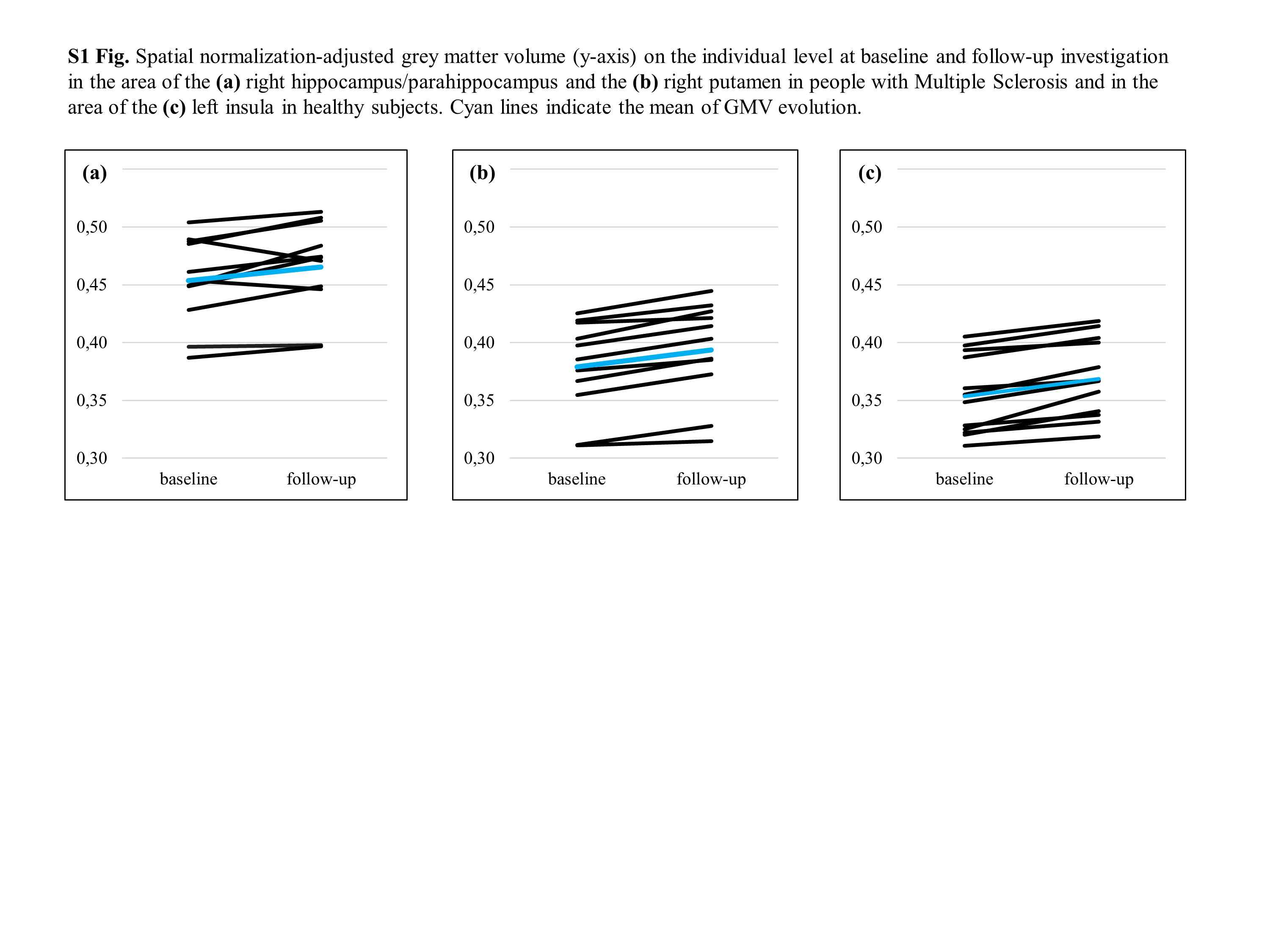

Supplement: S1 Fig — Spatial normalization-adjusted grey matter volume (y-axis) on the individual level at baseline and follow-up investigation in the area of the (a) right hippocampus/parahippocampus and the (b) right putamen in people with Multiple Sclerosis and in the area of the (c) left insula in healthy subjects. Cyan lines indicate the mean of GMV evolution. (TIF) [file pone.0226525.s003.TIF]

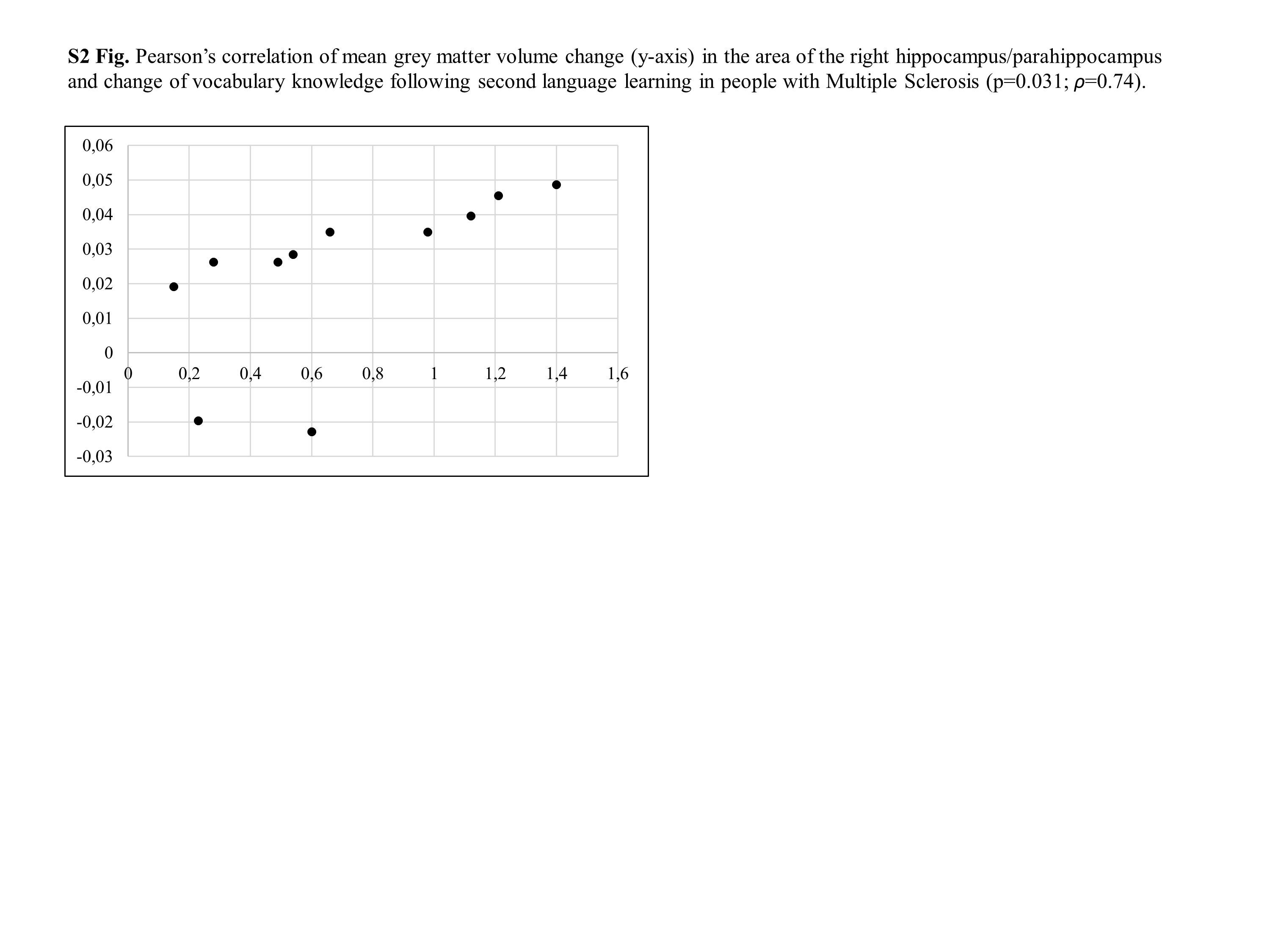

Supplement: S2 Fig — (TIF) [file pone.0226525.s004.TIF]
